# The Role of the Social Network in Access to Psychosocial Services for Migrant Elderly—A Qualitative Study

**DOI:** 10.3390/ijerph14101215

**Published:** 2017-10-11

**Authors:** Daphne Schoenmakers, Majda Lamkaddem, Jeanine Suurmond

**Affiliations:** 1 Department of Public Health, Academic Medical Centre/University of Amsterdam, 1012 WX Amsterdam, The Netherlands; d.h.schoenmakers@amc.uva.nl; 2 Kenniscentrum Sociale Innovatie, Lectoraat Toegang tot het Recht, Hogeschool Utrecht, 3584 CH Utrecht, The Netherlands; majda.lamkaddem@hu.nl

**Keywords:** ageing and diversity, access to health services, diversity health care provision, social network, ethnic minority, elderly migrants, health equity

## Abstract

**Background:** Despite high prevalence of mental problems among elderly migrants in The Netherlands, the use of psychosocial care services by this group is low. Scientific evidence points at the crucial role of social support for mental health and the use of psychosocial services. We therefore explored the role of social networks in the access to psychosocial care among elderly migrants in The Netherlands. **Methods:** A qualitative study was conducted using semi-structured group interviews and individual interviews. The eight group and eleven individual interviews (respectively n = 58 and n = 11) were conducted in The Netherlands with Turkish, Moroccan, Surinamese, and Dutch elderly. The data were analysed through coding and comparing fragments and recognizing patterns. **Results:** Support of the social network is important to navigate to psychosocial care and is most frequently provided by children. However, the social network of elderly migrants is generally not able to meet the needs of the elderly. This is mostly due to poor mental health literacy of the social network, taboo, and stigma around mental illness and the busy lives of the social network members. **Conclusions:** Strategies to address help-seeking barriers should consider mental health literacy in elderly migrants as well as their social networks, and counteract taboos and stigma of mental health problems.

## 1. Introduction

Apart from aging, international migration is one of the most influential factors that is transforming European demography. In Europe, around 10% of the elderly do not live in their country of origin and it is estimated that the number of elderly with a migration background will rapidly grow [1]. In The Netherlands, for example, 4% of the non-western migrants were 65 years and older in 2012 and it is expected that in 2050 this number will be increased to 18% of the non-western migrants [2]. This trend implies an increasing need for health care among elderly with a migration background. Most of them are first generation immigrants and, although there are differences between groups, many elderly migrants have low proficiency in the language of the host country and are often low educated with limited health literacy skills [3]. In addition, research indicates higher levels of mental illness in first generation migrants compared to the majority population [4,5,6]. Also, in The Netherlands, elderly non-western migrants more frequently suffer from common mental disorders such as anxiety, loneliness, and depression, compared to Dutch elderly [7,8].

Despite the fact that elderly migrants suffer more frequently from common mental disorders, the limited evidence shows that migrants in general make less use of psychosocial care services such as psychologists, social workers, and community centres [8,9,10,11,12,13]. Box 1 shows that psychosocial care in The Netherlands is accessible for all citizens but that access may be complex, for example because patients may be unaware of the possibilities of discussing mental health issues with their general practitioner (GP).

It is generally accepted that social support is essential for mental health [14] and having a social network is related to the use of psychosocial services [15]. A social network can be defined as the web of social relationships that surround an individual and which may be defined by, for example, size, boundedness, or frequency of contact [14]. Social networks, most often consisting of family members, relatives, or friends, are conduits of social support, i.e., these network members give emotional, informational, or instrumental support [14]. Emotional support refers to giving love and care, encouragement, and sympathy. Informational support is about providing information, facts, or advice that may help a person solve problems. Instrumental support refers to assisting with practical tasks [15]. 

Specifically, perceived emotional support is seen as positively influencing psychological well-being [14]. However, informational support (e.g., providing information about how to access psychosocial health care services) and instrumental support (e.g., bringing someone in the car to a health care provider) may also help in the access to psychosocial care services.

To date, there is limited and contradicting evidence about the role that social networks of elderly migrants play in the access to health care services. A Dutch study found that elderly migrants benefit from their social network (generally their daughters, sons, or daughters-in-law) who help them to navigate through the health care system by interpreting medical consultations between them and health care providers, by giving them advice about the health care system and by going along with them to appointments [16]. In addition, a study in the US found that elderly Mexican migrants involved their children as a resource for their mental health problems; they however, waited to seek psychosocial health services until mental health problems progressed to severe states [17]. A review also described that migrants tend to prefer seeking help from their social network, rather than to seek help from mental health services [18]. Another study in the UK described that the use of mental health services of Chinese migrants with mental illness was delayed because their social networks lacked the knowledge of mental health services [19,20]. 

Given these contradicting findings about whether the social network is enabling or inhibiting the access to psychosocial care service, as well as the fact that the role of the family and wider social networks in the access to psychosocial services is still unexplored in the Dutch context, the purpose of this study is to explore the role of the social network and potential ways of social support in access to psychosocial services for Turkish, Moroccan, and Surinamese elderly (the largest non-western ethnic minority groups in The Netherlands) and Dutch elderly.

Box 1 **Access to mental healthcare services in The Netherlands.**

Persons who experience mental health problems can visit a general practitioner (GP) or a community welfare worker. Community welfare workers are usually working from health centres, community centres, or the GP’s office. Persons with mild mental health problems usually can be treated by a GP often in cooperation with a general practice mental health worker. When the GP and/or the general practice mental health worker consider the mental health problems as too complex, they can refer either to a primary mental healthcare provider, or directly to secondary care. Primary mental healthcare providers treat mild to moderate mental health problems with treatment consisting of: counselling from a psychologist, psychotherapist, or psychiatrist; some form of online mental health support (e-health); a combination of counselling and online support. Patients with serious and complex psychiatric disorders, like anxiety disorder or post-traumatic stress disorder (PTSD), generally are treated in secondary mental health. Treatment is generally provided in a mental health institution, hospital, or private practice by a psychiatrist or clinical psychologist. Health insurance, which is compulsory for all citizens, covers all or part of the costs of primary and secondary mental health care with the exact conditions depending on the insurer and the policy.

## 2. Methods

### 2.1. Design 

This study was based on secondary data. The data was originally collected for a larger research project focusing on the use of several types of healthcare services among the largest groups of elderly migrants living in The Netherlands. For this project, both group interviews and individual interviews with elderly migrants and Dutch elderly were organized. The interviews focused on elderly suffering from self-defined physical limitations, loneliness, or depression. However, the group interviews focused on views of the respondents about other elderly sharing the same migration background and suffering from mental health problems. It did not specifically concern the respondents themselves. This choice was made in order to collect information about those elderly who actually never reach psychosocial care services. 

### 2.2. Recruitment 

All participants were orally approached using a community based recruitment method [21]. For the group interviews participants were recruited from community centres and church communities in Amsterdam and Utrecht. The groups were pre-existent natural groups and consisted of elderly with the same ethnicity. Regarding the individual interviews, potential participants were recruited with the help of community workers, care providers, or faith leaders. 

### 2.3. Data Collection 

Data collection took place in a period of 5 months, from May until September 2012. The group and individual interviews were semi-structured by using a topic-list. The interviews took 1 to 1½ h. All interviews were conducted in the preferred language (Dutch, Moroccan-Arabic, Berber, or Turkish) of the respondents with the assistance of trained bilingual interpreters. Interviews with the Surinamese respondents were conducted in Dutch, as their proficiency in Dutch is very good. Interviews were audiotaped, translated, and transcribed into Dutch by the interpreters. 

In total, eight group interviews (1 Dutch, 2 Moroccan, 3 Turkish, 2 Surinamese group interviews) with each between 3 and 10 participants were conducted. The participants of the group interviews were all aged 50 or above. In the group interviews, Turkish (*n* = 10) and Moroccan elderly women (*n* = 4) and Turkish (*n* = 17) and Moroccan elderly men (*n* = 7) participated. In one Surinamese group interview, 7 women from the Surinamese Creole ethnic group and 3 women from the Surinamese-Hindustani ethnic group participated. In the Dutch group interview, 4 women and 2 men participated. As a result of the community-based method of recruiting, the group interviews consisted of people who already know each other. This created overall a safe and supportive atmosphere where participants felt they could talk openly about the issues they felt were important. The group interviews were conducted in community centres or in a church: generally the places where these groups naturally meet. 

In addition, in the same period, eleven individual interviews were conducted. The respondents of the individual interviews were 60 years or older. For the individual interviews, in total 8 women and 3 men were interviewed. Respondents had a Turkish (*n* = 3), Surinamese (*n* = 3), Dutch (*n* = 3), or Moroccan (*n* = 2) background. The individual interviews were held with elderly people suffering from physical limitations, loneliness, or depression as defined by themselves. Individual interviews were conducted at the homes of the respondents, with the exception of one respondent who preferred to be interviewed in a community centre near his house.

For both the group interviews and the individual interviews, we stopped including new respondents when data saturation was reached.

### 2.4. Ethical Considerations

According to the Dutch Medical Research Involving Human Subjects Act, this research did not require medical-ethical approval. The medical ethical committee of the Academic Medical Centre confirmed this in writing on the 28th of October 2010. This study followed the ethical principles for medical research involving human subjects as laid down in the Declaration of Helsinki and adopted by the World Medical Association. In order to guarantee the anonymity of the participants, codes were used to designate the participants. Each participant was adequately informed of the aims and methods of the study. The participants gave a priori oral consent, which was audiotaped. 

### 2.5. Data Analysis

The interviews were translated to Dutch if necessary and all interviews were transcribed. The data was analysed by coding fragments, comparing observations, recognizing patterns, and structuring findings. In the first step an open coding strategy was applied. Close reading was used to split the interviews into different fragments, label relevant fragments, assign codes, and formulate memos and ideas [22]. In the next step a form of axial coding was used, which is a more abstract level of analysis [22]. This phase consisted of comparing fragments, determining relevance, and describing concepts. In this phase overarching themes emerged, which can be named as pattern codes [23]. Subsequently the fragments, ideas, reflections, and categories were structured by selective coding [22]. First, we analysed the interviews in terms of the social network that the respondents described, and we distinguished different types of network members (e.g., sons/daughters/daughters-in-law/spouses). In order to further organize the results, Excel-matrices were used. In these matrices results were ordered according to a framework that describes different consecutive stages of seeking and obtaining care and benefiting from the psychosocial services [24]. Roughly, this framework describes access to care in terms of the following consecutive stages: (1) the identification of needs; (2) seeking care services to address these needs; (3) the actual access to care services; and (4) the actual contact with a care provider. In addition, we ordered the results in terms of the type of perceived social support: emotional, instrumental, or informational support. We then described the patterns of the Excel-matrices and described the steps that individuals need to make in order to access psychosocial services, the type of social support given by the network members, and the type of network members that gave the social support. As we were interested in the social support given to elderly migrants, we will focus in the result section below on the interviews of elderly migrants and less on the interviews with Dutch elderly. 

## 3. Results

### 3.1. Social Network 

Nearly all the respondents (from all ethnic backgrounds) mentioned their children as an important tie within the social network. Overall, daughters seemed to be more important in the social network than sons, for example, they paid more visits to their parents’ house:

Interviewer: Do your children come?

Respondent: Yes, they regularly pay a visit. My daughters come three times a week.

Interviewer: Your sons don’t come?

Respondent: My sons rarely pay a visit.

[Moroccan women, group interview]

The role of spouses seemed limited: generally, respondents saw their spouse not as a source of social support, often because the health of the spouse was also compromised. Additionally, the role of friends was not large either, also because they often had mental health problems of their own and were not able to offer support:

I do not have stress, luckily. But I have many friends who do have stress. Because of issues with their children, the neighbours, they get depressed. We warn them not to get depressed, but still they do.

[Turkish man group, interview]

Thus, this part of the social network of elderly migrants is often frail and vulnerable, and cannot give social support because they are in need of support themselves.

The social network influenced the four consecutive steps in the help-seeking process towards psychosocial care: The social network influenced: (1) the identification of psychosocial needs of the migrant elderly; (2) the possibilities of seeking adequate psychosocial care services; (3) the possibilities of actual access to psychosocial care services; and (4) the actual contact with the psychosocial health care professional. We illustrate these steps in the following.

### 3.2. The Identification of Psychosocial Needs of the Migrant Elderly

In all the interviews the respondents mentioned either having mental health problems themselves or seeing others suffering from it. For example, in the Moroccan women group interview, all the respondents talked about having feelings of depression and loneliness themselves. The social network could play a large role in the identification and experience of mental health problems but often failed to do so. Firstly, elderly were not always able to recognize mental health problems, but their social network could not do so either; Secondly, if the social network did recognize mental health problems, it often did not allow elderly to talk about it with them; Thirdly, the social network caused its own stress and worries for the elderly. 

#### 3.2.1. The Social Network Does Not Recognize Mental Health Problems

Many respondents felt that it was difficult for elderly to recognize depressive feelings, as one of the participants explained about someone she knows:

That woman does not understand what a depression is. And her husband is depressive. (…) And she said, my husband has all the complaints (about depression) that you just mentioned. (…) And I said, if your husband is depressive than he should see a GP. He is ill, but his wife did not know it. (…) I said you should support him and take him to the GP. She said, and now I am depressive myself, I have the same complaints. So now they are both depressive.

[Moroccan woman, group interview]

Additionally, depressive feelings were seen as something that was part of life itself and as something normal:

All people have those feelings at time, that you feel depressed.

[Moroccan man; group interview]

The social network could often not support elderly migrants to recognize symptoms of depression, because they also missed the ‘mental health literacy’ to do so. 

#### 3.2.2. The Social Network Does Not Want Elderly to Talk about Their Mental Health Problems

The social network often could not support elderly migrants because members of the social network felt embarrassed or ashamed when elderly expressed mental health problems. Respondents of the Moroccan and Turkish women group for example described that their children often did not have sympathy for depressive parents. Their children generally had a very constraining influence on expressing mental problems and receiving help for mental problems. In the following fragment the respondents pointed out the unfavourable opinion of the children and their judging reaction when elderly expressed their mental health problems:

Respondent 1: They [the elderly] have to express their mental problems. It can happen to anyone, it is something that exists and has to be told. If you can’t tell a Turkish person, then you have to tell it a social worker. Ask them, what you have to do. But they don’t go, they don’t search for help.

Respondent 2: If your children hear it, they get angry. Why do you tell your problems, they say.

[Turkish woman, group interview]

Despite the fact that the social network often missed the mental health literacy to recognize symptoms of, for example, depression, or did not support the elderly to speak about their psychosocial needs, many respondents felt that talking about mental problems is important, as shown in the next fragment:

‘If you don’t express your problems, just not speak about your problems, then it will never heal.’

[Turkish woman, group interview]

#### 3.2.3. The Social Network Causes Its Own Stress and Worries for the Elderly

Several respondents indicated that social contact with their children helped against loneliness and depression but that a lack of contact with their children resulted in feelings of isolation. The respondents observed this both by themselves and by elderly in their social surroundings. Nevertheless, especially the Moroccan and Turkish group also expressed concerns about their children. The children caused a lot of stress, anger, worry, and wrangle, for example in the Moroccan female group interview the women worried about the marital status, truancy behaviour, and unemployment of children. According to the participants of the Turkish male group interview, westernization of the children led to cultural difference and this caused stress, as shown in the following fragment:

Respondent 2: There is a cultural difference between the children and us. We are not so well adapted here. And this causes stress.

[Turkish man, group interview]

As a consequence of this troublesome relationship, children were often not able to help their parents sufficiently. As a respondent explained:

We have to help our children, instead of them helping us.

[Turkish man, group interview]

### 3.3. The Possibilities of Seeking Adequate Psychosocial Care Services 

According to several respondents, some people who are suffering from mental illness will never seek help themselves. To help elderly with this, the social network generally offered some instrumental support, but often was not able to inform elderly about where to find help or did not have time to help elderly with this. In addition, some elderly were reluctant to discuss seeking help with their social network, because seeking help was seen as something for ‘mad people’.

#### 3.3.1. Social Network Offers Instrumental Support

Both migrant and Dutch elderly indicated that they depended on support from the social network when searching for care. In 3 group interviews, respondents (Dutch and Turkish) indicated explicitly that obtaining care themselves was too complicated. The required use of a computer was one of the complicating factors. Remarkable was the fact that Dutch elderly experienced in essence the same difficulties in arranging care compared to migrant elderly:

‘No, that’s something we don’t do anymore; we elderly people, the children have to do that. […] It’s just too complicated.’

[Dutch elderly, group interview]

Hence, all elderly experienced a lack of knowledge about how to access care, and their social network supported them, for example by helping with phoning, browsing on the Internet, and completing forms. For Turkish and Moroccan respondents, however, obtaining information was even more difficult because of linguistic barriers. While their children generally did not experience linguistic barriers, they still also did not have the knowledge about the Dutch health care system. Spouses also often were not seen as supportive either, for example, in the next excerpt the respondent tells that her husband does not know she is seeing a psychologist for her depression, because he would not understand what it entails:

*Interviewer: What does your husband think about the fact that you are seeing a psychologist?*

Respondent: Erm erm he does not know anything

Interviewer: Is that so? Why?

Respondent: He does not know what a psychologist is.

Interviewer: You do not talk about it?

Respondent: No no. We are not competent to talk to each other. We are not competent to fight nor to talk. We are quiet.

[Turkish individual interview]

#### 3.3.2. Social Network Does Not Have Time to Support Elderly 

Nearly all the group interviews and more than half of the individually interviewed elderly mentioned the busy lives of their children. According to several respondents, the children had many things to do and consequently did not have enough time to support their parents in the access to care:

The children do not help you. ‘Go by yourself’, they say. ‘You should take care of your own business’ they say. I have a lot of things to do’, they say.

[Turkish man, group interview]

The assumption that children are too busy was related to two factors. First, many respondents were afraid to be a burden to their children when they needed their help:

I make her mad, poor soul. My daughter. I constantly say: ‘do this, call him, do that, look there.’ And she needs time for studying too.

[Moroccan man, individual interview]

Second, elderly often had a wait-and-see attitude in contact with children. Nearly all the respondents said that their children have to take the initiative for face-to-face or phone contact. All the respondents explained this behaviour by referring to the diligent lives of their children or their sense of pride.

‘I say no, I don’t even consider it, you (children and grandchildren) have to call me, I don’t have to call you!’

[Surinam woman, group interview]

#### 3.3.3. Psychological Help Is for ‘Mad People’

Some respondents argued that they were reluctant to find help for their depressive complaints because psychological help was seen as something for ‘mad people’:

I said, I am not going there (psychologist). Mad people go there. (…) I am not a mad person.

[Turkish men, group interview]

Elderly with mental health complaints did not always ask their social network to help them with searching for psychosocial help, because of taboos around seeing a psychologist. 

### 3.4. The Possibilities of Actual Access to Psychosocial Care Services

According to most respondents, the social network helped them with actually getting to the psychosocial care services. The social network offered instrumental support and arranged appointments, facilitated transport to care services, or gave information about transport facilities and locations of psychosocial care services. The role of children is well illustrated in the following fragment. Here, a Turkish woman explained which function the children fulfilled in access to social care (community centre):

The children have to bring their mother one or two times. As usual, she will soon get used to it when she meets friendly people and has nice conversations. That’s the reason they say: lovely talks and friendly faces will get a snake out of his hole. When she has this positive experience, she will go by herself. Then she will not wait until her children will bring her. She will come by herself. That’s the way it goes.

[Turkish woman, individual interview]

While children are especially important to initiate contacts with social care, positive experiences with social care will help the elderly to go by themselves after a while. 

Emotional support, however, was sometimes more difficult to obtain from the social network. A few female elderly indicated that their husbands had a constraining influence. For example, husbands prohibited them to go to a community centre. The situation of a friend of the respondents was discussed in this fragment:

She is just not allowed [by her husband] to go with us to the community centre.

[Turkish women, group interview]

### 3.5. The Actual Contact with the Psychosocial Health Care Professional

One Turkish respondent with mental health problems was seeing a Turkish psychologist, and was relieved that she could speak in Turkish about her problems. However, other Turkish and Moroccan respondents experienced linguistic barriers in the contact with care providers. Consequently, Turkish and Moroccan elderly depended on the availability of their social network to translate:

Interviewer: What kind of difficulties do you experience?

Respondent 1: Always, for example, I don’t speak Dutch. When I need an appointment, I have to arrange it according to the agenda of my children. Therefore I can’t go to an appointment in the morning.

Respondent 2: Indeed, you have to write it down in their agenda, when you don’t speak the language.

Respondent 1: And the children have to go to work, so they can only arrange it when they have no other obligations.

[Turkish women, group interview]

Respondents from other migrant groups (Surinamese Creole and Surinamese Hindustani) did not encounter language barriers, as their proficiency in Dutch is generally good.

## 4. Discussion

In our study, the social network affected different steps in access to psychosocial care for migrant elderly: Firstly, the social network often could not help migrant elderly with recognizing mental health problems, either because they lacked the knowledge of symptoms or out of shame; Secondly, the social network often could not help migrant elderly with searching for psychosocial health either because elderly did not want to burden them, because they also lacked the knowledge where to find it or because looking for psychological help was a taboo; Thirdly, the social network offered instrumental and informational support for elderly to visit psychosocial health care services, but often was not able to provide emotional support; And fourthly, when elderly migrants actual were in contact with a care provider, they were dependent on their social network (e.g., children) for translation, but this also interfered with the agenda of the children. 

Hence, while many elderly migrants experience mental health problems, many of them never reach psychosocial health services, because they fail to navigate through all the steps and their social network is not able to support them in this. One reason for this were the linguistic barriers experienced by Turkish and Moroccan elderly which made them more dependent on their children for navigating through all the steps to reach psychosocial health services. Another reason was the low mental health literacy of elderly migrants, but also of their network. Symptoms of mental health problems were not always recognized and treated by the network with shame and as a taboo.

Several other studies also stressed that the help-seeking process involves a number of steps. For example, in one study participants would first try to resolve their mental health problems on their own, would then seek help from their social network if this was ineffective, and only as a last resort would seek help from psychosocial services [19]. However, in our study, the social network was involved in all steps, but could not provide adequate support to reach psychosocial services either. While migrant elderly lacked mental health literacy and the language skills to reach psychosocial services, their children oftentimes also did not have the knowledge about how and where to find psychosocial services, as was also noted in other studies [25,26,27]. More importantly, as we found in our study, their dismissive attitude towards the expression of depressive feelings or other expressions of distress, was not supportive in the help-seeking process of the elderly migrants. As a previous review found, seeking help from formal services was often associated with shame and stigma [19], however, in our study, not only by the elderly themselves, but also by their social network.

Out of fear to encumber their children, many elderly were hesitant to ask children for social support. This fear of being a burden to the children is in large contrast with the ‘stereotypical idea’ of care providers who often assume that the family will provide care for the elderly migrants. This ‘discourse of looking after their own’ as Peckover and Chidlaw [28] have put it, has said to influence care providers who refrain from offering care services, leaving elderly migrants and their carers isolated and unsupported [28,29,30,31,32,33]. While it is often believed that for elderly migrants a social network is crucial and that they tend to use informal networks instead of professional help for psychological problems, we would like to argue that elderly migrants often do not have the adequate resources in their social network.

### Strengths and Limitations

The combination of group interviews and individual interviews seemed to be a useful way to explore the role of social networks in the access to care services in migrant and Dutch elderly. We created a safe environment during the interviews by using bilingual interpreters and by using known contact persons of the elderly. Even though this group is hard to reach, we succeeded in reaching a hard to reach group and elicited their perspectives on the social network and the access to psychosocial care services.

In most group interviews, respondents indicated that children regularly provided insufficient social support, which was a far more negative opinion than observed in the individual interviews. Group dynamics may have influence on the things said in the group interviews and could therefore be a limitation of this study. In contrast, in the individual interviews, respondents may have felt inhibited to talk negatively about the children in front of the interviewer. By complementing the group interviews with the individual interviews, we believe that we have triangulated the data and have obtained more reliable data. 

Another limitation is the small amount of interviews per ethnic group, therefore, no firm conclusions about differences between ethnic groups could be made. It was, however, not our intention to investigate or point out the differences between ethnic groups. We aimed to explore the role of social network in elderly migrants in general and Dutch elderly. We did not include other migrant groups in The Netherlands, such as Chinese migrants, nor did we include elderly refugees. These other migrant groups may have different experiences with the social support from their social network in the access to psychosocial health services. We, however, believe that also in these migrant groups linguistic barriers and low mental health literacy will cause barriers for the elderly and will ask specific skills from their social network to support them in this.

Current Dutch policies are focused on decreasing healthcare expenditure and stimulating low cost care based on community care. This implies that citizens are stimulated to take care of each other and, as a consequence, more is expected from the social network. In the context of the transition to low cost care based on community care in The Netherlands, the inability of the social network to support properly must be taken into account. Educating the social network and the community may increase mental health literacy, and may empower the social network as well as the elderly themselves, to find psychosocial health services for mental health needs. Eventually, migrant elderly are expected to benefit because better insights will likely lead to improved access to psychosocial care and more successful care provision. 

In addition, service developers should consider how services might be adapted to meet the needs of clients from migrant groups. For instance, verbal and visual information may be given rather than written information [34], meeting the elderly needs related to low levels of education and low levels of literacy in their birth language. Health information may be better targeted to different language and literacy needs. 

While the social network often feels responsible for interpreting and translating tasks, at the same time the social network may filter information given to the elderly, especially when sensitive information or issues around stigma have to be translated [35]. This also suggests that information is tailored not only to the needs of the social network but also to the needs of elderly migrants themselves. Furthermore, psychosocial services should involve trained interpreters when needed, rather than to leave this task to the social network of the elderly migrants [35]. This not only may relieve the feelings of elderly of being a burden to their children, but also may give them the opportunity to ask and receive information by themselves, rather than that their always dependent on their children for information.

Finally, health care services can be more outreaching, rather than waiting for clients to come. Our study showed that elderly migrants may not have a clear question for help, and, for example, see depression as something that belongs to life. Feelings of shame may also impede elderly from asking health care providers for help. GPs, but also district nurses or home care providers may be more aware that elderly will not ask easily for help for their mental health problems and may be more proactive. Training can focus on how to ask elderly migrants about their mental health problems and help them to identify whether further help is needed. Also, the social network may be approached proactively, by asking them about the possible burden they experience or by giving advice about where to find help.

## 5. Conclusions

This study contributes to a better understanding of the role of the social network in access to psychosocial care. The results show on the one hand that elderly migrants depend on support from their social network and on the other hand that they often are confronted with the inadequacy of their social network. Members of the social network regularly lacked health literacy, competences, or time to support the elderly in access to care. 
